# Cost-effectiveness of reflex laboratory-based cryptococcal antigen screening for the prevention and treatment of cryptococcal meningitis in Botswana

**DOI:** 10.12688/wellcomeopenres.15464.2

**Published:** 2020-03-13

**Authors:** Mark W. Tenforde, Charles Muthoga, Andrew Callaghan, Ponego Ponatshego, Julia Ngidi, Madisa Mine, Alexander Jordan, Tom Chiller, Bruce A. Larson, Joseph N. Jarvis

**Affiliations:** 1 University of Washington School of Medicine, Seattle, Washington, 98195, USA; 2 University of Washington School of Public Health, Seattle, WA, 98195, USA; 3 Botswana-UPenn Partnership, Gaborone, Botswana; 4 Botswana Harvard AIDS Institute Partnership, Gaborone, Botswana; 5 National Health Laboratory, Gaborone, Botswana; 6 Centers for Disease Controls and Prevention, Atlanta, Georgia, 30329-4018, USA; 7 Boston University School of Public Health, Boston, MA, 02118, USA; 8 London School of Hygiene & Tropical Medicine, London, UK

**Keywords:** Cryptococcal antigen, CrAg, HIV, AIDS cost-effectiveness, modelling

## Abstract

**Background: **Cryptococcal antigen (CrAg) screening for antiretroviral therapy (ART)-naïve adults with advanced HIV/AIDS can reduce the incidence of cryptococcal meningitis (CM) and all-cause mortality. We modeled the cost-effectiveness of laboratory-based “reflex” CrAg screening for ART-naïve CrAg-positive patients with CD4<100 cells/µL (those currently targeted in guidelines) and ART-experienced CrAg-positive patients with CD4<100 cells/µL (who make up an increasingly large proportion of individuals with advanced HIV/AIDS).

**Methods: **A decision analytic model was developed to evaluate CrAg screening and treatment based on local CD4 count and CrAg prevalence data, and realistic assumptions regarding programmatic implementation of the CrAg screening intervention. We modeled the number of CrAg tests performed, the number of CrAg positives stratified by prior ART experience, the proportion of patients started on pre-emptive antifungal treatment, and the number of incident CM cases and CM-related deaths. Screening and treatment costs were evaluated, and cost per death or disability-adjusted life year (DALY) averted estimated.

**Results: **We estimated that of 650,000 samples undergoing CD4 testing annually in Botswana, 16,364 would have a CD4<100 cells/µL and receive a CrAg test, with 70% of patients ART-experienced at the time of screening. Under base model assumptions, CrAg screening and pre-emptive treatment restricted to ART-naïve patients with a CD4<100 cells/µL prevented 20% (39/196) of CM-related deaths in patients undergoing CD4 testing at a cost of US$2 per DALY averted. Expansion of preemptive treatment to include ART-experienced patients with a CD4<100 cells/µL resulted in 55 additional deaths averted (a total of 48% [94/196]) and was cost-saving compared to no screening. Findings were robust across a range of model assumptions.

**Conclusions: **Reflex laboratory-based CrAg screening for patients with CD4<100 cells/µL is a cost-effective strategy in Botswana, even in the context of a relatively low proportion of advanced HIV/AIDS in the overall HIV-infected population, the majority of whom are ART-experienced.

## Introduction

Cryptococcal meningitis (CM) is a leading cause of mortality in people living with HIV/AIDS (PLWH) worldwide, causing an estimated 15% of HIV deaths ^1^. HIV-associated CM predominantly occurs in the setting of advanced HIV disease, typically at a CD4 T-cell count <100 cells/µL ^2^. In patients with advanced HIV initiating antiretroviral therapy (ART), detection of cryptococcal antigen (CrAg) in the blood is highly predictive of subsequent CM ^3^, with clinical symptoms usually developing within a few weeks ^3, 4^. CrAg screening with pre-emptive fluconazole therapy in CrAg-positive ART-naïve adults (without symptoms/signs of CM) has been shown to reduce all-cause mortality and is recommended by the World Health Organization (WHO) in adults starting ART with a CD4 <100 cells/µL ^5, 6^.

Botswana, a country of approximately 2.3 million with a 2017 adult HIV prevalence of 23% ^7^, has a mature HIV program providing free ART to citizens since 2002. Despite ART scale-up, advanced HIV/AIDS and CM remain common with almost 400 confirmed cases diagnosed per year in 2013 and 2014 ^2, 8, 9^. In 2016, national HIV guidelines first recommended CrAg screening in ART-naïve, CrAg-positive patients with a CD4 <100 cells/µL ^10^. For CrAg-positive ART-naïve patients, fluconazole 1200 mg/day for 2 weeks, then 800 mg/day for 8 weeks with ART initiation, then 200 mg/day until CD4 recovers to >200 cells/µL for at least 6 months is recommended. Currently, “reflex” screening (screening of any sample sent for CD4 testing below the CD4 count threshold) is conducted at the Botswana-Harvard HIV Reference Laboratory (BHHRL), which performs most CD4 testing in the urban Gaborone region. The cost-effectiveness of CrAg screening has not been evaluated in Botswana.

In addition, with reflex CrAg screening, a new question has arisen: how to manage ART-experienced, CrAg-positive patients. With a laboratory-based, reflex CrAg screening program, a CrAg test is performed following all CD4 results <100 cells/µL. Since the lab does not know who is treatment-naïve and who is not, both ART-naïve and -experienced patients are CrAg screened. Previous models assumed that all or most CrAg-screened patients are ART-naïve ^11– 13^, and therefore only focused on ART-naïve patients. However, in Botswana as well as elsewhere in sub-Saharan Africa, over half of CM cases now occur in ART-experienced patients ^14– 16^, and reflex screening where CD4 monitoring is conducted is now identifying an important number of ART-experienced and CrAg-positive patients. This population represents a mix of individuals: 1) those recently started on ART without baseline CD4 and CrAg screening results following adoption of the HIV “test-and-treat” strategy ^17^; 2) those on ART but with treatment failure; and 3) those started on ART with subsequent ART default re-engaging in ART care. Current CrAg screening guidelines do not address management of this growing population, although there is a highly plausible benefit of treating these CrAg-positive patients.

 The primary aim of our study was to model the cost-effectiveness of CrAg screening and targeted pre-emptive fluconazole treatment in Botswana using different screening and treatment policies. A decision analytic model was developed based on prior models ^13, 18^, with local CD4 distribution ^8^, CrAg prevalence ^19^, treatment outcomes ^14^, ART status, and costing data. Two related policies are evaluated. For Policy 1, based on current guidelines, we modeled reflex CrAg screening for any patient with a CD4 <100 cells/µL and then pre-emptive treatment only for the ART-naïve patients ^10^. The model for Policy 1 is used to estimate national costs for screening and the cost-effectiveness of this screening policy compared to no screening based on the cost per death averted and the cost per disability-adjusted life year (DALY). For Policy 2, we extend the analysis of Policy 1 to also incorporate pre-emptive treatment for ART-experienced individuals identified as part of reflex screening. We evaluated these models under a range of assumptions.

## Methods

### Overview

Our models use CD4 count distribution and CrAg prevalence data from the BHHRL in Gaborone, including data from a completed CrAg screening prevalence study conducted 2015–2016 and a second cohort from January 2018 through January 2019 ^19^. Both studies received ethical approvals from the Botswana Health Research and Development Committee (HRDC) [HPDME 13/18/1] and the University of Pennsylvania Institutional Review Board (#827814), along with this cost-effectiveness analysis. CrAg testing is performed using the highly accurate CrAg lateral flow assay (LFA) [IMMY, Norman, OK] ^20^, assumed for the analysis to be 100% sensitive and specific. Approximately 65,000 CD4 tests are performed annually at the BHHRL in 35,000 unique patients. With an estimated population of 370,000 adults in Botswana living with HIV, most of whom know their HIV-status and have engaged with HIV-care services ^21, 22^, this covers nearly 10% of the adult HIV-positive population in Botswana. We therefore chose to start with a model of 650,000 CD4 tests performed annually on a population of 350,000 adults to provide an estimate of the annual cost and impact of CrAg screening implementation at a national level. Our models include two components: 1) A reflex CrAg screening module; and 2) a treatment module.

### Screening module for the current policy (Policy 1)

**Figure 1.**
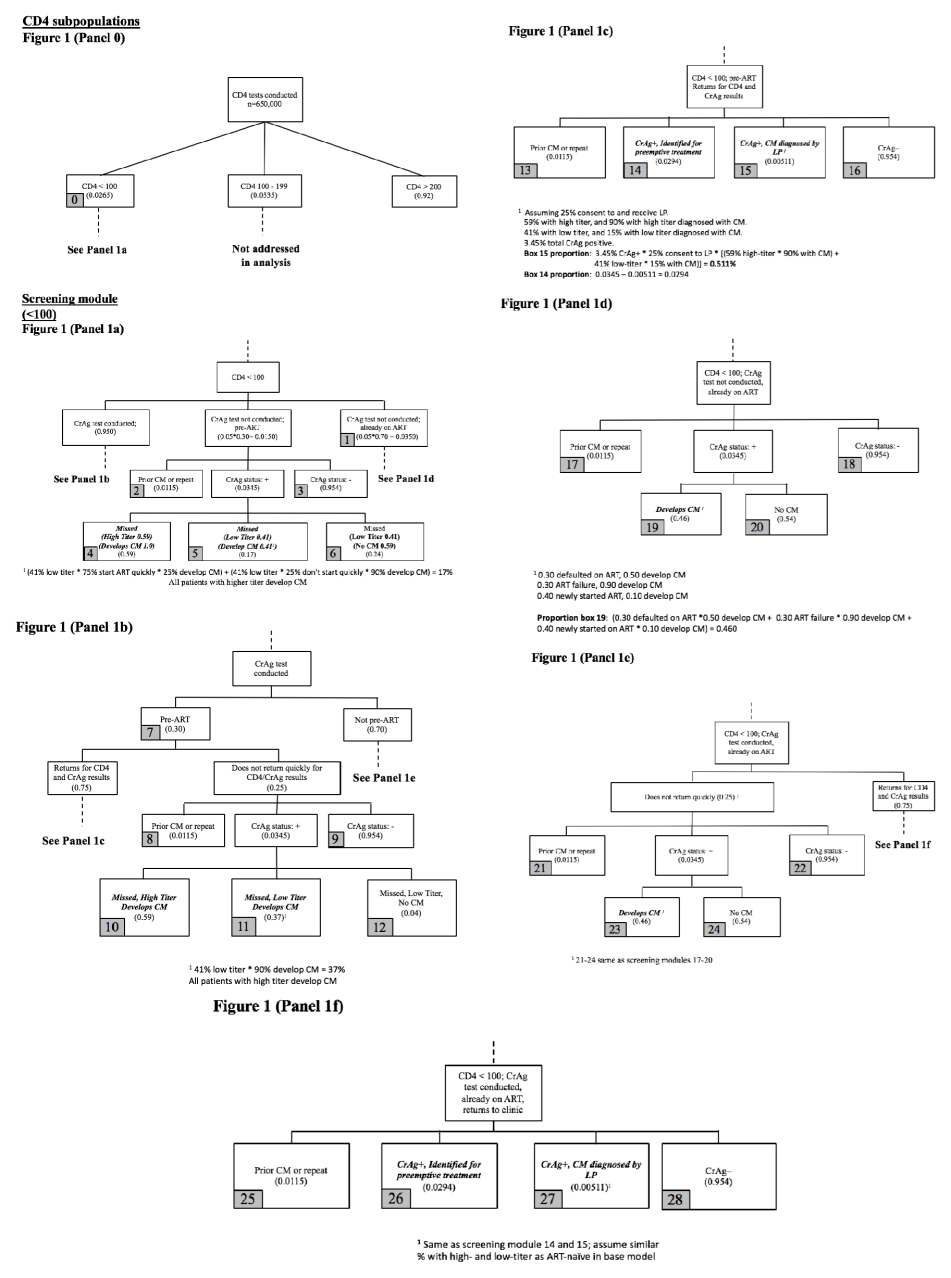
Flowcharts of screening module. Panel **0** describes the proportion of CD4 tests with a CD4 <100 cells/µL (target population for CrAg screening per national Botswana guidelines). ( **a**) Outcomes for pre-ART, CrAg-positive patients eligible for screening but in whom screening is not conducted. ( **b**) Outcomes for pre-ART patients who screen CrAg-positive but do no return for urgent follow-up. ( **c**) Outcomes for pre-ART patients who screen CrAg-positive and return for urgent follow-up. ( **d**) Outcomes for ART-experienced, CrAg-positive patients eligible for screening but in whom screening is not conducted. ( **e**) Outcomes for ART-experienced patients who screen CrAg-positive but do not return for urgent follow-up. ( **f**) Outcomes for ART-experienced patients who screen CrAg-positive and return for urgent follow-up.

The screening model estimates the proportion of patients who receive CD4 testing with a CD4 count <100 cells/µL, the proportion with prior evidence of ART use, the proportion CrAg-positive, the proportion who are preemptive treatment-eligible, and the proportion of these patients who remain asymptomatic and are targeted for preemptive therapy or are diagnosed with CM on urgent clinic follow-up after a positive CrAg test. The model includes imperfect “real-world” implementation, so that a proportion of eligible patients may not receive CrAg screening or are screened but do not receive timely evaluation. Figure 1 summarizes the screening model for Policy 1 (CrAg screening if CD4 <100 cells/μL), and Table 1 provides sources for parameter estimates. A full description of model estimates and data sources is available as *Underlying data* ^23^.

**Table 1.** Key parameters, estimates, and sources of data for base model.

**Screening Module**		
Parameter	CD4 <100 cells/μl	Source(s)
% within CD4 strata	2.65%	BHHRL data
CrAg prevalence within CD4 strata (outpatient), %	4.6%	19, 24
Prior CM among screened CrAg+, %	25%	19
High CrAg titer (≥1:180), %	59%	19
Pre-ART of CrAg+, %	30%	Local cohort ^8^
Return quickly enough of CrAg+, %	75%	Assumption
Treatment Module		
Parameter	CD4 <100 cells/μl	Source(s)
Hospitalized if missed CrAg+ and develops CM, %	80%	Assumption
10-week CM mortality	50%	14
CM relapse	17%	14
High CrAg titer and fail pre-emptive therapy (if receive fluconazole)	20%	25, 26
Low CrAg titer and fail pre-emptive therapy (if receive fluconazole)	5%	25, 26
Hospitalized if fail pre-emptive therapy and develop CM	90%	Assumption
10-week mortality	25%	25
CM relapse	17%	14
Hospitalized if diagnosed with CM at urgent follow-up visit	100%	Assumption
10-week mortality	25%	25
CM relapse	17%	14

BHHRL = Botswana-Harvard HIV Reference Laboratory; CM = cryptococcal meningitis

In Figure 1, 2.65% of CD4 samples from clinics have a CD4 count <100 cells/µL (based on estimates from 2015–2017), with a local CrAg prevalence of 4.6% at CD4 <100 cells/µL in non-hospitalized patients ^19^. Only 30% of patients have no prior viral load testing documented in the national electronic medical record (EMR), Integrated Patient Management System. As baseline viral load testing is not performed in Botswana, with initial viral load testing 3–6 months after starting ART ^10^, any client with viral load testing prior to CrAg screening can be assumed to be ART-experienced. Further, of CrAg-positive samples approximately 25% are assumed to be from patients with prior treated cryptococcal disease, which results in a persistently positive test result. These patients are not targeted for pre-emptive therapy. The remaining 75% of CrAg-positive patients are potentially eligible for pre-emptive fluconazole.

We used local CrAg titer data to estimate the risk of ART-naïve CrAg-positive patients progressing to CM ^19^. A high CrAg titer (>1:160) is associated with a high risk of CM without pre-emptive treatment in ART-naïve patients ^25, 26^. From local data, 59% of CrAg-positive patients with a CD4 <100 cells/µL have a high CrAg titer. A lumbar puncture is offered to all CrAg-positive patients evaluated for pre-emptive therapy to rule out prevalent CM, as up to one-third of even relatively asymptomatic CrAg-positive patients have prevalent meningitis diagnosed when evaluated by lumbar puncture (LP) and cerebrospinal fluid (CSF) testing ^25, 26^. As LP refusal is common ^27^, we estimate that only 25% of patients consent to an LP. A total of 85% of high-titer and 15% of low-titer patients who undergo LP are diagnosed with prevalent CM and hospitalized ^25^. The remaining CrAg-positive patients (i.e. those who did not undergo LP or had negative CSF testing) are targeted for pre-emptive fluconazole.

A proportion of CrAg-positive patients who should receive CrAg screening are not tested (5%), e.g. due to laboratory error or CrAg assay stockout. Without CrAg testing and pre-emptive fluconazole, these patients are at an high risk of incident CM, with risk influenced by a patient’s CrAg titer and how quickly ART is initiated ^3, 25, 28^. Of screened CrAg-positive patients, an estimated 25% with CD4 <100 cells/µL do not return to clinic quickly for evaluation and initiation of pre-emptive fluconazole and/or LP evaluation; these patients are also at a high risk of progression to CM.

### Treatment module for the current policy (Policy 1)

**Figure 2.**
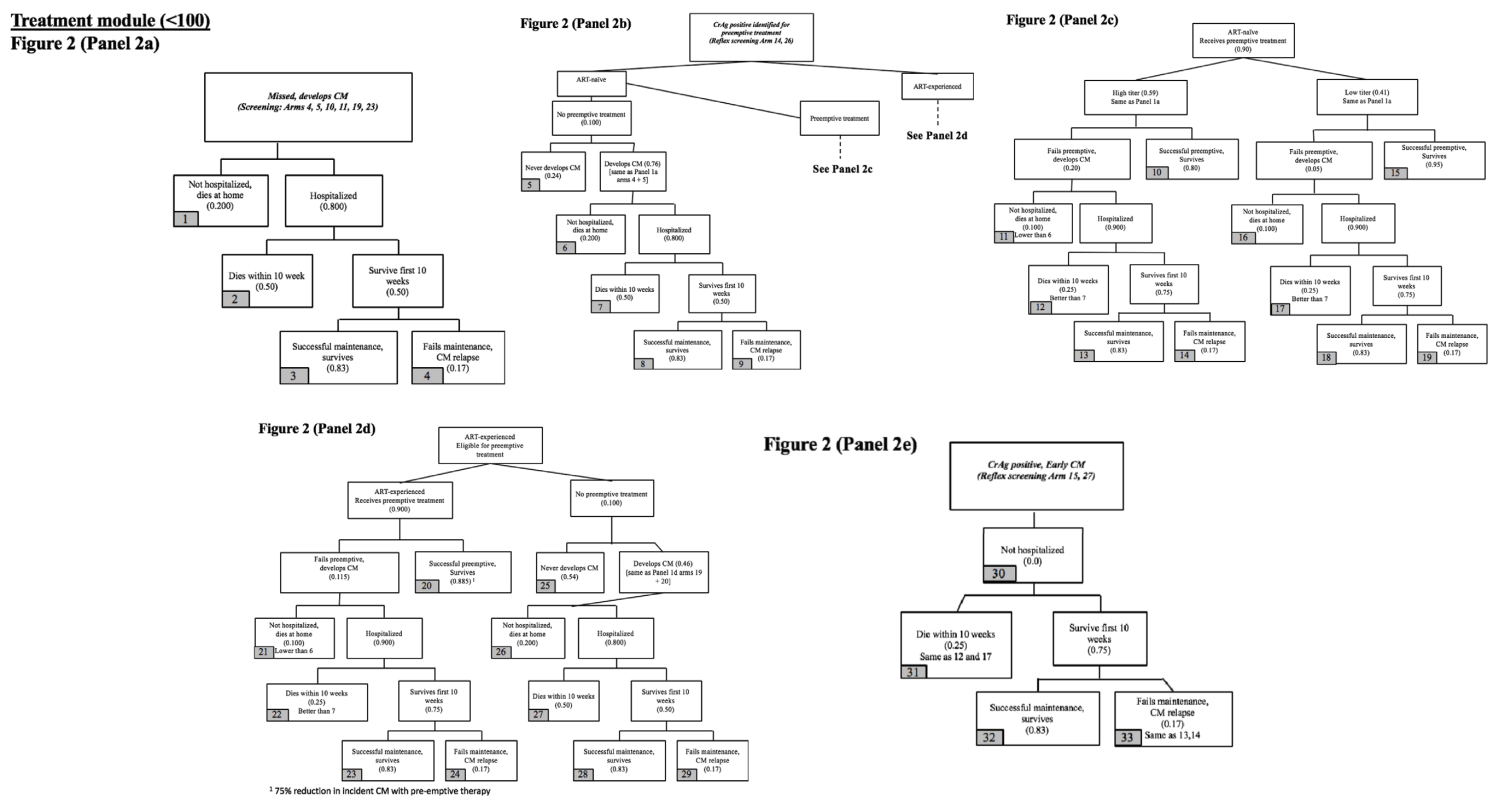
Flowcharts of treatment module. ( **a**) Outcomes for CrAg-positive patients with missed CrAg screening who develop cryptococcal meningitis. ( **b**) Outcomes for ART-naïve, CrAg-positive patients who are identified for pre-emptive treatment but do not receive it. ( **c**) Outcomes for ART-naïve, CrAg-positive patients who receive pre-emptive treatment. ( **d**) Outcomes for ART-experienced, CrAg-positive patients who receive or do not receive pre-emptive treatment. ( **e**) Outcomes for CrAg-positive patients diagnosed with cryptococcal meningitis on urgent follow-up.

The treatment module estimates outcomes for (a) patients who should receive CrAg screening but do not and progress to CM, (b) those who receive screening but progress to CM without urgent follow-up and pre-emptive therapy, (c) those diagnosed with CM at the initial urgent follow-up visit, and (d) those who start but “fail” pre-emptive treatment, developing CM. Flowcharts for the treatment module are shown in Figure 2, and sources for estimates detailed in the *Underlying data* ^23^.

For ART-naïve CrAg-positive patients, outcomes of pre-emptive treatment are derived from local estimates and a published systematic review ^19, 28^; we estimate that 20% of high-titer patients and 5% of low-titer patients started on pre-emptive fluconazole will fail pre-emptive fluconazole and go on to develop CM.

For patients who progress to CM, case-fatality rates are derived from local data ^14^. Ten-week mortality for patients with a CD4 <100 cells/µL hospitalized for cryptococcal meningitis and treated with amphotericin B-based induction therapy is estimated to be 50%, with 17% of survivors experiencing relapse. CrAg-positive patients who are diagnosed with early CM on urgent follow-up or hospitalized after failing pre-emptive fluconazole are assumed to have a lower 10-week mortality (25% instead of 50% for those missed and not started on pre-emptive treatment) based on regional data ^25^, with early recognition of infection and timely initiation of antifungal treatment associated with better survival. In our models, we assume that a minority of patients (20%) with incident CM die at home without being diagnosed, with a lower proportion who start on pre-emptive fluconazole (10%) or are diagnosed with CM at their initial follow-up (0%).

### Screening and treatment modules extending treatment to ART-experienced CrAg-positive clients (Policy 2)

In Policy 2, in addition to ART-naïve patients we model preemptive treatment for ART-experienced CrAg-positive patients with a CD4 <100 cells/µL. From 2018–2019 cohort data, approximately 70% of patients with a CD4 <100 cells/µL had documented HIV viral load testing by the time of CrAg screening indicating a history of ART use. Some of these ART-experienced patients have experienced ART failure or default and are assumed to be at high risk for progression to CM, whereas others are recently started on ART with HIV viral suppression awaiting immune recovery and assumed to be at comparatively lower risk for progression to CM. For CrAg-positive patients with ART treatment failure, without preemptive fluconazole therapy we assume a 90% risk of progression to CM based on anticipated delays in ART regimen change and prolonged immunosuppression. For CrAg-positive patients who have defaulted ART and are now re-engaging in care, we assume a 50% risk of progression to CM without preemptive fluconazole therapy, or approximately twice as high as the published risk of CM progression in CrAg-positive ART-naïve patients who do not receive preemptive therapy ^3, 28^. We estimate a higher risk compared to ART-naïve patients starting ART because of a greater likelihood of worse adherence or default in this population with previous ART default. For patients who recently started on ART with good virological response who are awaiting CD4 recovery, we assume a 10% risk of progression to CM without preemptive therapy, about half the risk in CrAg-positive ART-naïve patients starting ART who do not receive preemptive therapy ^3, 28^. As the median time to CM diagnosis is approximately 5 weeks in CrAg-positive patients newly starting ART without preemptive therapy and incidence of CM falls rapidly following ART initiation ^3, 4^, this estimate accounts for the fact that these patients have already been on ART for several weeks or months without a diagnosis of CM and therefore are likely at a lower risk of progression.

From 2018–2019 local cohort data, 40% of ART-experienced patients with a CD4 <100 cells/µL had a suppressed HIV viral load within 3 months prior to the date of CrAg screening and are assumed to fit into this lower-risk category (10% for CrAg-positive patients without preemptive therapy). The remaining 60% without recent HIV viral load testing or a recent non-suppressed HIV viral load are assumed to be at higher risk for CM progression due to ART default (50% risk in CrAg-positive without preemptive therapy) or treatment failure (90% risk in CrAg-positive without preemptive therapy). About half of these patients (30%) had no viral load testing in the previous 6 months and are assumed to have defaulted, whereas the other half (30%) had viral load testing within 6 months but with their last viral load unsuppressed and are assumed to have experienced treatment failure.

 For ART-experienced patients, given lack of published outcomes data in this population we assume that there is a 75% reduction in the risk of progression to CM with pre-emptive therapy. In the base model, we also assume that the same proportion of ART-experienced clients who are seen at urgent follow-up are diagnosed with CM by lumbar puncture as with ART-naïve clients. Screening and treatment flow diagrams are shown in Figure 1 and Figure 2 for Policy 2.

### CrAg screening and treatment unit costs

The costs of CrAg screening implementation and CM treatment are estimated from a provider-perspective using 2018 local supply costs from Botswana Central Medical Stores (CMS) for most parameter estimates ( Table 2 and *Underlying data* ^23^). Costs in Botswana Pula were converted to United States dollars (US$) using mid-2018 exchange rates (10.61 Pula to US$1, which has remained largely unchanged). For CrAg screening, the cost per CrAg test is estimated at 50 Pula (US$4.71); to the wholesale price (~US$2), we factor in additional mark-ups from local distributors, shipment, and laboratory personnel costs. Fluconazole is relatively expensive in Botswana (at ~US$0.50 per 200 mg tablet through CMS) compared to other countries in the region and we assumed procurement through CMS rather than a pharmaceutical company drug donation program. Average length of maintenance therapy is estimated at 6 months, assuming some incomplete adherence.

**Table 2.** Included cost estimates for CrAg screening and pre-emptive treatment and for cryptococcal meningitis treatment.

CrAg screening and pre-emptive therapy *		
Parameter	Estimate (USD)	Source(s)
CrAg LFA	$4.71	IMMY wholesale plus additional costs
Pre-emptive fluconazole 1200 mg/day x2 weeks 800 mg/day x8 weeks 200 mg/day x26 weeks	$0.51 / 200 mg tablet x 490 tablets = $247.54	CMS; proportion with treatment failure or partial adherence
Urgent return evaluation	$18.85	Assumption
Treatment Module *		
Parameter	Estimate (%)	Source(s)
Hotel costs 17-day hospital stay	$188.51 / hospital day	14, 29
Hospital drug and procedure costs Including 14 days AmBd and FLU, 2 lumbar punctures	$202.24 (survives), $151.68 (dies)	CMS; 14
Post-admission costs FLU consolidation/maintenance, Extra clinic visit	$226.37	CMS
Laboratory costs 2 FBC, 4 U/E, 1 ALT	$71.00	BHHRL; 6

* See Supplementary Excel File for detailed costing estimates ALT = alanine aminotransferase; AmBd = amphotericin B deoxycholate; BHHRL = Botswana Harvard HIV Reference Laboratory; CM = cryptococcal meningitis; CMS = Central Medical Stores; FBC = full blood count; FLU = fluconazole; KCl = potassium choloride; Mg = magnesium supplementation; NS = normal saline; U/E = urea and electrolyte testing; WHO = World Health Organization

We do not have reliable local hospital “hotel” costs in Botswana for the treatment of CM. Therefore, WHO-CHOICE estimates for cost per day of hospital admission in 2008 are used assuming most cases are managed at referral/teaching hospitals with 100% occupancy and 10,000 admissions per year, and inflation-adjusting for 2018 prices using International Monetary Fund estimates ^2, 29, 30^. Length of hospital stay was estimated at 17 days for survivors for local data (75% of this time in those who die within 10 weeks) ^14^. We assume a standard 14-day course of amphotericin B with high-dose fluconazole, intravenous fluid and electrolyte supplementation, and routine laboratory monitoring. Patients who survive hospitalization receive 8 weeks of fluconazole consolidation and 6 months of maintenance fluconazole on average. Patients who die within 10 weeks of treatment have lower utilization of treatment, laboratory, and post-discharge care and fluconazole (see *Underlying data* ^23^).

### Outcomes

We estimate total annual costs of CrAg screening and treatment (both for pre-emptive fluconazole and cryptococcal meningitis) from the provider perspective for each policy. Comparing these to a counterfactual scenario with no screening, we estimate the number of cryptococcal meningitis cases averted from screening, number of deaths prevented, and the cost per death averted as the incremental cost effectiveness ratio. We also evaluated cost per DALY avoided. With an average age of death from CM of 36 years from local data ^14^, age-specific, gender-averaged life expectancy of 36 additional years from 2016 WHO Global Health Observatory data ^31^, and with a 3% annual discount rate, we estimated 21.4 DALYs avoided per death avoided. We then re-evaluated models with both ART-naïve (the target population for CrAg screening) and ART-experienced CrAg-positive patients being offered pre-emptive treatment. We do not factor in additional costs or deaths from CM relapse as these are small and unlikely to have a significant public health or health system cost impact.

### Sensitivity analyses

We performed a series of sensitivity analyses to account for areas of uncertainty or possible changes in CD4 testing practices. As Policy 2 dominated no CrAg screening and Policy 1 (see Results), all sensitivity analyses were performed for Policy 2:

Sensitivity analysis 1: This model assumes 50% (versus 75% in the base model) of CrAg-positive patients return quickly for urgent follow-up and initiating of pre-emptive therapy with other parameter estimates remaining the same.

Sensitivity analysis 2: This model assumes a combined 25% risk of CM progression for CrAg-positive ART-experienced patients without pre-emptive therapy (versus 46% in the base model) and with no CrAg-positive ART-experienced patients diagnosed with CM by lumbar puncture at urgent follow-up. Other parameter estimates remain unchanged. This sub-analysis accounts for uncertainty in the benefit of preemptive therapy for CrAg-positive ART-experienced patients given the lack of published literature in this group.

Sensitivity analysis 3: This model assumes 70% of clients are ART-naïve and 30% ART-experienced (versus 30% and 70%, respectively, in the base model) with risk of progression to CM with and without pre-emptive therapy unchanged. The sub-analysis was conducted to account for potential changes in CD4 testing practices.

## Results

### Cryptococcal meningitis cases and costs without screening

Without CrAg screening, our base model estimates 17,225 CD4 counts <100 cells/µL. In the base model without CrAg screening, 305 patients with a CD4 <100 cells/µL develop CM without screening, 41% (126/305) are ART-naïve, and 196 CM-related deaths occur, with an additional 22 relapse CM cases ( Table 3). The number of cases is lower than the nearly 400 CM cases microbiologically confirmed annually in Botswana without CrAg screening ^2^; however, a proportion of patients will be diagnosed and hospitalized with CM without recent CD4 testing. The proportion of patients who are ART-naïve is consistent with local and regional estimates that half or more CM cases now occur in ART-experienced individuals ^14, 15^. The overall total cost for CM treatment is estimated at $851,716,

**Table 3.** Estimated cryptococcal meningitis cases, deaths, and costs without CrAg screening.

Population: CD4 < 100 cells/μL	Results - ART- naïve		Results - ART- experienced		Results - Total	
	Number patients	Cost for patients	Number patients	Cost for patients	Number patients	Cost for patients
Identified for preemptive treatment (but did not receive), but did not develop CM -- survives	0	0	0	0	0	0
Identified for preemptive treatment, receives treatment, survives	0	0	0	0	0	0
Not hospitalized, dies	27	0	38	0	65	0
Hospitalized, dies < 10 weeks	54	148,992	77	210,661	131	359,653
Hospital, survives maintenance	45	169,191	64	239,221	109	408,413
Hospital, CM relapse	9	34,654	13	48,997	22	83,651
Total Treatment Costs		352,837		498,879		851,716
Total Screening Costs		0		0		0
Total Costs		352,837		498,879		851,716
Total Cases	135		191		326	
Total Deaths	81		115		196	

ART = antiretroviral therapy; CM = cryptococcal meningitis; CrAg = cryptococcal antigen

### Policy 1. CrAg screening at CD4 <100 cells/µL, ART-naïve only

In our CD4 <100 cells/µL base model, 16,364 CrAg tests are performed at a cost of $77,073. Without screening, we estimate 196 CM-related deaths annually in this CD4 <100 cells/µL population. For ART-naïve only, with CrAg screening and treatment, we estimate 39 lives saved (a 20% reduction in CM-related deaths among those patients with CD4 testing), at a cost of $43 per death prevented or $2 per DALY averted ( Table 4).

**Table 4.** Estimated cryptococcal meningitis cases, deaths, and costs for CrAg screening and treatment of only ART-naïve (Policy 1).

Population: CD4 < 100 cells/μL	Results - ART- naïve		Results - ART- experienced (same as above with no screening)		Results - Total	
	Number patients	Cost for patients	Number patients	Cost for patients	Number patients	Cost for patients
Identified for preemptive treatment (but did not receive), but did not develop CM -- survives	3	0	0	0	3	0
Identified for preemptive treatment, receives treatment, survives	84	22,351	0	0	84	22,351
Not hospitalized, dies	12	60	38	0	51	60
Hospitalized, dies < 10 weeks	30	82,847	77	210,661	107	293,508
Hospital, survives maintenance	38	142,912	64	239,221	101	382,133
Hospital, CM relapse	8	29,271	13	48,997	21	78,268
Total Treatment Costs		277,440		498,879		776,320
Total Screening Costs		77,073		0		77,073
Total Costs		354,513		498,879		853,393
Total Cases	88		191		279	
Total Deaths	42		115		157	

ART = antiretroviral therapy; CM = cryptococcal meningitis; CrAg = cryptococcal antigen

### Policy 2. CrAg screening at CD4 <100 cells/µL, both ART-naïve and ART-experienced

We next considered treating both ART-naïve as well as ART-experienced patients recognized as CrAg-positive through reflex screening. No additional costs are accrued for screening ART-experienced patients. Under base model assumptions, an additional 55 lives were saved through treatment of ART-experienced patients ( Table 5). Treatment of both ART-naïve and ART-experienced resulted in an overall savings of $1421 per death averted, or $66 per DALY averted. Policy 2 dominated both no screening and the Policy 1 strategy of treatment only for ART-naïve clients ( Table 6). CrAg screening under Policy 2 remained cost-saving across a range of scenarios (see *Underlying data* ^23^ for results of sensitivity analyses).

**Table 5.** Estimated cryptococcal meningitis cases, deaths, and costs for CrAg screening and treatment of both ART-naïve and ART-experienced (Policy 2).

Population: CD4 < 100 cells/μL	Results - ART- naïve		Results - ART- experienced		Results - Total	
	Number patients	Cost for patients	Number patients	Cost for patients	Number patients	Cost for patients
Identified for preemptive treatment (but did not receive), but did not develop CM -- survives	3	0	14	0	16	0
Identified for preemptive treatment, receives treatment, survives	84	22,351	201	53,574	285	75,925
Not hospitalized, dies	12	60	16	116	28	176
Hospitalized, dies < 10 weeks	30	82,847	44	130,742	73	213,588
Hospital, survives maintenance	38	142,912	64	150,503	102	293,415
Hosptal, CM relapse	8	29,271	13	28,486	21	57,757
Total Treatment Costs		277,440		363,421		640,861
Total Screening Costs		77,073		0		77,073
Total Costs		354,513		363,421		717,934
Total Cases	88		137		225	
Total Deaths	42		59		102	

ART = antiretroviral therapy; CM = cryptococcal meningitis; CrAg = cryptococcal antigen

**Table 6.** Incremental cost-effectiveness ratio for CrAg screening and treatment strategies.

Population: CD4 < 100 cells/μL	Deaths	Costs	Change costs	Change deaths	DALY averted	Cost per death averted	Cost per DALY averted	Comments
No screening	196	851,716	n/a	n/a	n/a	n/a	n/a	Initial comparison policy
Base Model: Screening < 100, preemptive txt only ART-naïve	157	853,393	1,676	-39	829	43	2	Dominates no screening
Base Model: Screening < 100, preemptive txt both ART-naïve and ART-experienced	102	717,934	-133,782	-94	2014	-1421	-66	Dominates Policy 1 (compared to preemptive txt only ART naïve)

## Discussion

Using robust local clinical, outcomes, and costing data, we provide the first estimates of the cost-effectiveness and impact of CrAg screening implementation in Botswana. This analysis was intended to be pragmatic, reflecting current CD4 testing practices in real world settings in Botswana, a country where national guidelines currently recommend CD4 testing at baseline (pre-ART), at three months, at 12 months and yearly thereafter for stable clients ^10^. This strategy, coupled with adoption of HIV “test-and-treat” ^32^, contributes to a growing proportion of ART-experienced CrAg-positive individuals recognized through laboratory-based reflex screening in Botswana. Existing screening guidelines and modeling studies have not considered the impact of this large population or benefits of pre-emptive treatment. As in other studies from sub-Saharan Africa ^11, 13, 18^, we found CrAg screening in ART-naïve patients with a CD4 <100 cells/µL to avoid a DALY for a low cost (e.g., substantially less than one year of ART medication costs). Targeting only CrAg-positive ART-naïve patients for pre-emptive therapy, however, had a modest public health impact, preventing only 20% of CM-related deaths under base model assumptions. Additionally, treating CrAg-positive ART-experienced patients resulted in additional deaths prevented, and was cost-saving or avoided a DALY for a very low cost across a range of assumptions.

CD4 testing remains necessary for identifying individuals with advanced HIV disease (i.e. low CD4 count) and guiding CrAg screening and other preventive measures against common opportunistic infections. With decreased funding for CD4 testing and an increasing focus on HIV viral load rather than CD4 monitoring in patients on ART ^17^, our findings may be less relevant in countries that do not support CD4 assessment following ART initiation. Nevertheless, in settings where post-ART CD4 testing is available, CD4 monitoring and CrAg screening—particularly in individuals with treatment failure or a history of default—may provide an additional public health benefit as the HIV epidemic matures in sub-Saharan Africa with a majority of cryptococcal meningitis occurring in ART-experienced individuals. International guidelines should address the clinical management in this emerging population, and future outcomes research is needed to better inform the benefit of preemptive therapy.

Our study has a number of limitations. First, we had significant uncertainty for a number of parameter estimates, particularly the risk of progression to cryptococcal meningitis for CrAg-positive individuals previously started on ART and the proportion of clients identified as CrAg-positive who receive urgent follow-up care in real-world settings. To account for uncertainty, we performed a series of sensitivity analyses demonstrating that CrAg screening avoids DALYs for a low cost, even with lower risk of CM progression in ART-experienced individuals or relatively poor urgent clinical follow-up. We also had robust local estimates for most cost and clinical parameters, particularly CD4 distribution, CrAg prevalence, and CM treatment outcomes data to support our findings ^14, 19^. Secondly, as a pragmatic study using real-world data, we did not evaluate other screening strategies such as point-of-care CD4 and/or CrAg testing, and focused on CrAg screening in clinic settings without considering a potential benefit in hospitalized patients ^33, 34^. Thirdly, our analysis may fail to account for future changes in CD4 testing practices in Botswana, including a potential shift away from CD4 testing as in other settings ^17^. Finally, we limited our analysis to CrAg screening in patients with a CD4 count <100 cells/µL, reflecting current national guidelines. With the WHO recently conditionally recommending CrAg screening in individuals with a CD4 count of 100–200 cells/µL ^6^, further analysis is needed to model the impact of CrAg screening and preemptive therapy in this patient population with a lower CrAg prevalence ^24^.

In summary, using local estimates and accounting for uncertainty through sensitivity analyses, we found strong support for CrAg screening in Botswana for patients with advanced HIV at a threshold of <100 cells/µL. In addition to preemptive therapy in ART-naïve individuals, our findings provide support for pre-emptive treatment of CrAg-positive ART-experienced patients at a CD4 <100 cells/µL recognized in laboratory-based reflex CrAg screening programs. Future outcomes data is needed for this growing population and guidelines should consider the evidence for pre-emptive treatment in this group.

## Data availability

### Underlying data

Open Science Framework: Cost effectiveness of cryptococcal antigen screening in Botswana. https://doi.org/10.17605/OSF.IO/URXAG ^23^.

This project contains the underlying data used for this modeling study, including a description of all estimates and their sources under tab “Screening Parameter Estimates”.

Data are available under the terms of the Creative Commons Zero "No rights reserved" data waiver (CC0 1.0 Public domain dedication).
