# Supplementary material for: Cost-effectiveness of reflex laboratory-based cryptococcal antigen screening for the prevention and treatment of cryptococcal meningitis in Botswana
Source: Wellcome Open Res. 2020 Mar 13;4:144. Originally published 2019 Sep 25. [Version 2] doi: 10.12688/wellcomeopenres.15464.2 (PMC6871359; doi:10.12688/wellcomeopenres.15464.2)
Supplement: Peer review history

# Peer review: Reviewer response for version 1

**DOI:** 10.21956/wellcomeopenres.16911.r37060

**Published:** 2019-11-18

**Authors:** Felix Bongomin

**Affiliations:** 1 Department of Medical Microbiology and Immunology, Faculty of Medicine, Gulu University, Gulu, Uganda

WHO recommends CrAg screening for all PWH with CD4 count <100. A few studies from resource-constrained settings have shown CrAg screening to be cost-effective and is associated with improved survival among patients with advanced HIV disease.

 In the present study, the cost-effectiveness analysis accounted for CrAg screening and pre-emptive azole therapy in both ART-naive and ART-experienced PWH in Botswana - reflecting a real-world situation in Sub-Saharan Africa. 

 The assumptions used in the modelling are backed-up by local data.

 However, the authors seemingly overestimated the LP refusal rate (75%, vs 12-30% in published data from SSA). In effect, the estimated number of CM cases was lower than expected.

I confirm that I have read this submission and believe that I have an appropriate level of expertise to confirm that it is of an acceptable scientific standard.

# Response

**Published:** 2020-02-03

**Authors:** Mark Tenforde

**Affiliations:** University of Washington, Seattle, USA

Thank you for these comments. Our estimate for the proportion of patients who screen CrAg-positive and consent to lumbar puncture is based on local data in Botswana. In a recent CrAg-screening cohort in which CrAg-positive patients were evaluated in clinic by a study doctor and nurse and asked to consent to lumbar puncture evaluation for meningitis, less than 40% on enrolled patients consented to lumbar puncture. In real-world settings without a dedicated study team, we assumed that consent to lumbar puncture evaluation would be lower.

# Peer review: Reviewer response for version 1

**DOI:** 10.21956/wellcomeopenres.16911.r36577

**Published:** 2019-10-10

**Authors:** Deborah K. Glencross

**Affiliations:** 1 Department of Molecular Medicine and Haematology, Faculty of Health Sciences, University of Witwatersrand, Johannesburg, South Africa; 2 National Health Laboratory Services, Johannesburg, South Africa

Tenforde *et al. *have investigated the cost-effectiveness of reflexed laboratory-based CrAg screening based on CD4 counts <100 cells/μl. The model used is based on the assumption that 75% of patients will return for their CD4 count within days, and that 90% of these patients receive care for CM. Realistically, indeed based on evidence and knowledge of loss-to-follow-up (LTFU) rates in South Africa, the percentage of patients who return for treatment and care (in this instance, to get their CD4 counts) is lower than the 75% assumed for this study. Assuming that 90% of patients with identified CrAg-positivity are enrolled for follow-up and receive care, may also be optimistic.

 Politicians and/or policymakers will use/act on the outcomes of such cost-effectiveness studies to decide whether (or not) to implement a widescale programme of care. In a resource poor setting, with a high burden of advanced HIV disease, such as seen throughout Southern Africa, such consideration is crucial to ensure the best use of limited resources. A best-case scenario could lead to unrealistic expectations, at lower cost expectations, especially if baseline assumptions, including the number of people who return for care and/ or who receive treatment, are higher than actual numbers. The model presented by Tenforde *et al.* could therefore be considered a near best-case scenario outcome.

 This is not so much a criticism of this work but more a comment on the practical value of cost-effectiveness work. Although it is important to present a scenario that will encourage policymakers to adopt, in this instance, widescale reflexed CrAg screening, policymakers may appreciate being presented with less-favourable outcomes too, so that they could additionally consider outcomes in a worst-case scenario. This approach would enable an understanding of how the overall outcome could be affected if fewer patients return for care before making any decisions about undertaking widescale implementation. The authors could therefore consider including some additional modelling and cost-effectiveness outcomes about a worst-case scenario.

 In my opinion, a worst-case review doesn’t detract from the expectation that the programme will be implemented. Rather it acts to emphasise to policymakers that systems need to be fully effective and in place for the programme to succeed and be cost-effective. It is an opportunity to identify possible challenges (and perhaps, where the programme is likely to fail and not be cost-effective) so that problems can be dealt with beforehand. Thereafter, the best-case scenario model can be realised and the best-case cost-effectiveness that is predicted will prevail.

I confirm that I have read this submission and believe that I have an appropriate level of expertise to confirm that it is of an acceptable scientific standard.

# Response

**Published:** 2020-02-03

**Authors:** Mark Tenforde

**Affiliations:** University of Washington, Seattle, USA

We appreciate these comments from Dr. Glencross. As the reviewer states, in real-world settings a lower proportion of cryptococcal antigen (CrAg)-positive patients may return for urgent evaluation following CrAg screening.

The baseline model that we present assumes successful national implementation of CrAg screening and pre-emptive treatment. We performed an additional sensitivity analysis (Sensitivity Analysis 1) assuming that only 50% rather than 75% of screened CrAg-positive patients return for an urgent follow-up evaluation. Under this assumption, the cost of CrAg screening of only ART-naïve patients per disability-adjusted life year (DALY) averted was USD 138, with screening for both ART-naïve and ART-experienced being cost-saving. We concluded that screening implementation was likely still cost-effective even under less optimistic assumptions.
